# Psychosocial issues need more attention in COPD self-management education

**DOI:** 10.1080/02813432.2020.1717087

**Published:** 2020-02-06

**Authors:** Hannele Siltanen, Tiina Aine, Heini Huhtala, Marja Kaunonen, Tuula Vasankari, Eija Paavilainen

**Affiliations:** a Department of Health Sciences, Faculty of Social Sciences, Tampere University, Tampere, Finland;; b Nursing Research Foundation, Helsinki, Finland;; c The Finnish Centre for Evidence-Based Health Care: A Joanna Briggs Institute Affiliated Group, Helsinki, Finland;; d Faculty of Medicine and Health Technology, Tampere University, Tampere, Finland;; e Pirkanmaa Hospital District, Tampere, Finland;; f Division of Medicine, Department of Pulmonary Disease and Clinical Allergology, University of Turku, Turku, Finland;; g Finnish Lung Health Association (FILHA), Helsinki, Finland;; h Etelä-Pohjanmaa Hospital District, Seinäjoki, Finland

**Keywords:** COPD, patient education, self-management, specialised healthcare, primary healthcare, asthma/COPD nurses

## Abstract

**Objective:** To find out how regularly the contents of patient education regarded as essential for COPD patients’ self-management are provided by healthcare professionals in specialised healthcare (SHC) and primary healthcare (PHC) in Finland.

**Design:** A cross-sectional study based on an e-questionnaire with 42 items on the content of self-management education of COPD patients.

**Setting:** The study sample included all public SHC units with pulmonary outpatient clinics (*n* = 29) and nine out of 160 health centres in Finland.

**Subjects:** 83 doctors and 162 nurses.

**Main outcome measures:** The respondents’ answers on how regularly they included the contents regarded as essential for COPD patients’ self-management in their education of COPD patients.

**Results:** COPD patients were educated regularly on medical issues regarding COPD treatment, such as smoking cessation, exercise and pharmacological treatment. However, issues vital for coping with the disease, such as psychological well-being, stress management or fatigue, were often ignored. Patient education in SHC seemed to be more systematic than education in PHC. The education provided by the asthma/COPD nurses (*n* = 70) was more systematic than the education provided by the other nurses (*n* = 84).

**Conclusion:** Healthcare professionals’ continuous education should cover not only the medical but also the psychosocial aspects of coping with COPD. The role of doctors and nurses should be considered to ensure that there is no gap in COPD patients’ education. Training asthma/COPD nurses and promoting specialised nurse-led asthma/COPD clinics in primary care could be beneficial while improving practices of patient education that enhance patients’ ability to cope with the disease.

**KEY POINTS**
- Issues vital for coping with chronic obstructive pulmonary disease (COPD), such as psychological well-being, stress and fatigue, are irregularly included in self-management education both in primary and specialised healthcare.
- Patient education provided by asthma/COPD nurses is more regular than patient education provided by other nurses.
- The distribution of work between doctors and nurses should be considered to ensure that there is no gap in COPD patients’ education.

## Introduction

Chronic obstructive pulmonary disease (COPD) is one of the leading causes of mortality and morbidity worldwide [1]. Smoking is the main causative factor of COPD, and it is a common risk factor for a variety of co-morbidities, such as cardiovascular diseases, osteoporosis and musculoskeletal diseases [2]. COPD patients also have other co-morbidities, including depression and anxiety [3].

Smoking cessation, adequate use of medication, regular physical exercise and healthy nutrition are the cornerstones of treatment of COPD. The aim of treatment is to relieve symptoms, promote quality of life, delay progression, prevent exacerbations and reduce mortality [4].

COPD patients need information and education to successfully perform self-management. An ability and the motivation to perform self-management increases their quality of life and protects them from exacerbations. Thus, patient education should be included as an essential part of COPD treatment [5]. Self-management is a lifetime task in which patients need to develop skills, such as problem-solving, decision-making, resource utilisation, taking action and developing a patient–healthcare provider partnership [6]. Furthermore, patients have to learn how to integrate those skills in their everyday life, thereby enabling certain behavioural changes, such as smoking cessation [7]. A prerequisite for an effective alliance between COPD patients and healthcare professionals is that healthcare professionals are familiar with the essential themes of patient education. The themes are global and the same for all COPD patients, irrespective of their healthcare organisation or country.

According to the Finnish national guidelines [8], primary healthcare (PHC) is mainly responsible for the early detection and diagnosis of COPD, and only certain COPD patients are treated in specialised healthcare (SHC). Doctors in PHC are mostly specialists in general practice or unspecialised doctors, whereas doctors in SHC usually are pulmonologists or registrars. Nurses in PHC and in SHC can be registered nurses, specialised nurses (e.g. in internal or surgical diseases) or public health nurses. Furthermore, they can achieve a special competence in asthma/COPD. However, the education has not been systematically organised, and it has not been a vital prerequisite for working as an asthma/COPD nurse. In Finnish PHC, a COPD patient usually meets a doctor and, sporadically, a nurse. This depends on the patient’s situation and the healthcare organisation’s arrangements. In SHC, patients usually visit both a doctor and a nurse.

The aim of this study is to find out how regularly the contents of patient education regarded as essential for COPD patients’ self-management in the literature are provided by healthcare professionals, both doctors and nurses, in practical work in SHC and PHC in Finland.

## Materials and methods

### Survey questionnaire

The questionnaire was developed by two authors of the research (HS and EP) utilising previous studies [9–12], clinical pathways [13] and guidelines [8], and it was pretested for this cross-sectional descriptive study. The e-questionnaire comprised 42 items concerning the contents of patient education regarded as essential for COPD patients’ self-management together with questions pertaining to the background of the respondent, such as age, experience of COPD treatment and whether the respondent was working as an asthma/COPD nurse (Supplementary file).

### Research subjects

The link to the e-questionnaire was mailed to chief physicians and head nurses of all pulmonary disease departments in the university hospitals (*n* = 5) and district hospitals (*n* = 24) as well as to chief physicians and head nurses of primary health centres (*n* = 9 out of 160 centres) in Finland. The study sample included all public SHC units with pulmonary outpatient clinics in the country, with the exception of Swedish-speaking Ahvenanmaa. Furthermore, a sample of nine PHC centres was selected as a representative sample based on their size (two small and seven large centres) and location around Finland.

The chief doctors and head nurses were asked to forward the link for the e-questionnaire and the study information sheet to their subordinates, that is, doctors and nurses who took care of COPD follow-up visits. The survey was carried out between 1 October 2016 and 15 December 2016.

### Analysis

**Figure 1.**
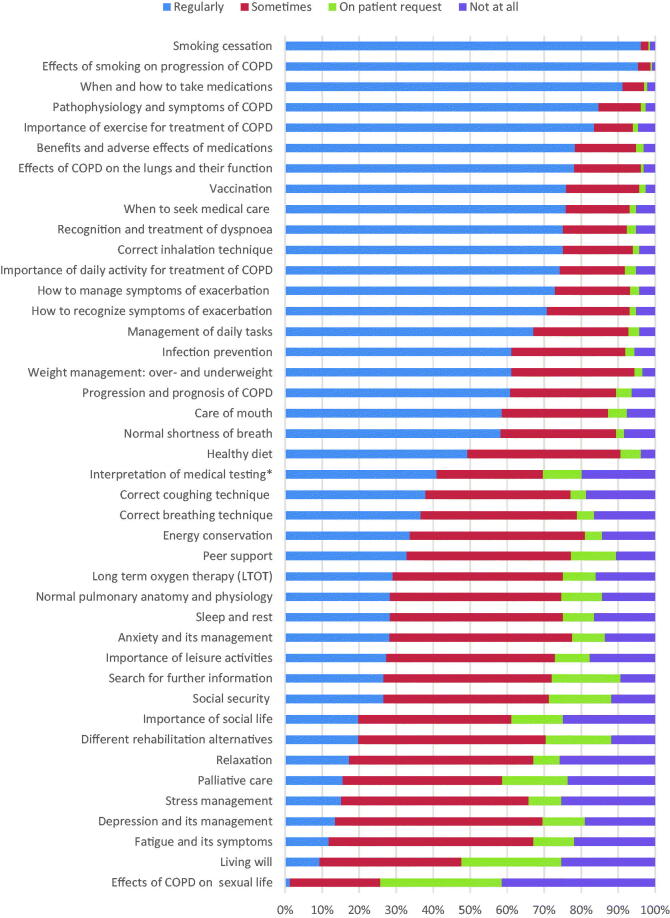
The regularity of self-management education provided by doctors and nurses in PHC and in SHC (*n* = 233). *Interpretation of the results of spirometry and other physical measurements.

In the questionnaire, the respondents were asked how often on a general level they included each of the 42 educational topics in their education with COPD patients. The response options were education is provided ‘regularly’, ‘sometimes’, ‘on patient request’ or ‘education is not provided at all’. In the analysis, the first category, named ‘regularly’, has been seen as an indicator of the established practice in the organisation, whereas all the other alternatives have been seen as representing an action that could be coincidental and could include a risk of non-education. Thus, the results described below are based on the ‘regularly’ responses, with the exception of Figure 1.

Data analysis was performed using SPSS version 22.0. Descriptive statistics are presented as frequencies and percentages.

## Results

In total, 245 completed questionnaires were returned. The respondents from SHC units (*n* = 27/29 units, 93%) included 49 doctors and 79 nurses, and the respondents from health centres (*n* = 9/9) included 34 doctors and 79 nurses. There were 4 nurses who did not identify their workplace. The answers of 8 nurses (5 from SHC, 3 from PHC) were excluded due to their special job descriptions. Thus, the final data included 83 doctors and 154 nurses. The mean age of the doctors was 46.6 years (range 24–66) and of the nurses 45.2 years (range 22–63). Their mean experience of COPD treatment was 17.3 years (range 0.5–38) and 11.8 years (range 0.5–39), respectively. Almost half of the nurses worked as an asthma/COPD nurse (45.5%, *n* = 70) and of these, 56.7% (*n* = 38) worked in SHC.

In the whole study population, the best covered content areas of COPD self-management education included medical issues regarding COPD treatment, such as smoking cessation, pharmacological treatment and exercise (Figure 1). However, issues related to coping with the disease, such as stress, depression and fatigue, as well as end-of-life decisions, including palliative care and a living will, were often poorly covered.

**Figure 2.**
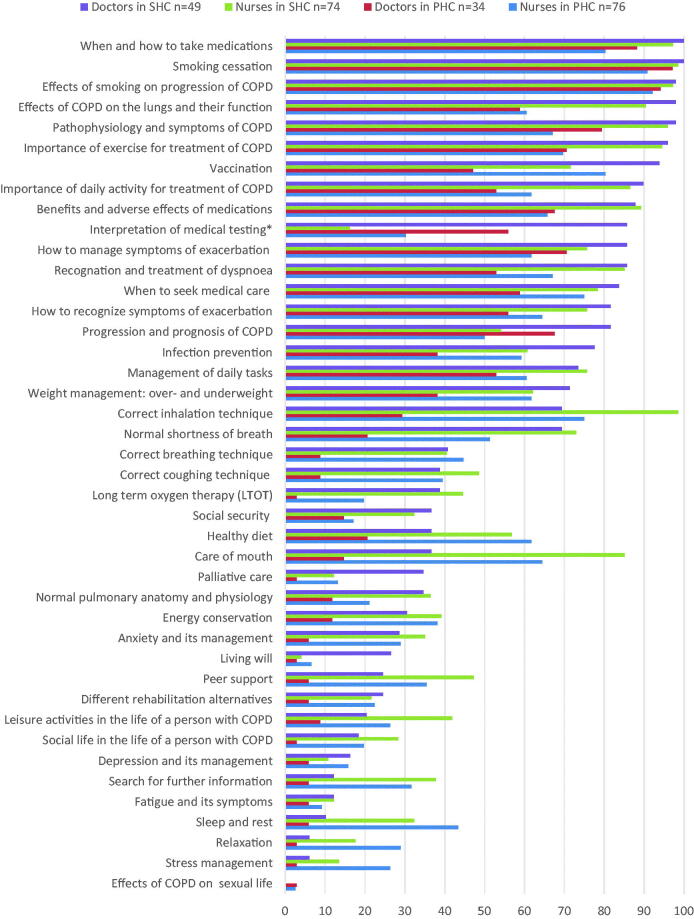
The regularity of self-management education provided by doctors (*n* = 49) and nurses (*n* = 74) in SHC and doctors (*n* = 34) and nurses (*n* = 76) in PHC. *Interpretation of the results of spirometry and other physical measurements.

In general, doctors and nurses emphasised different content areas in their education of COPD patients (Figure 2). Doctors educated more often on the diagnostic procedures and treatment options of COPD, whereas nurses were more active on the practical management of COPD treatment, for example, correct inhalation technique, mouth care or nutrition. As a whole, the nurses were responsible for a wider range of COPD education topics than the doctors, especially in PHC but also in SHC.

COPD education was provided more regularly in SHC than in PHC in almost all the content areas, and that applied to both doctors and nurses (Figure 2). Regarding the poorly covered psychosocial aspects of COPD education, nurses, especially those in PHC, were more active than doctors.

**Figure 3.**
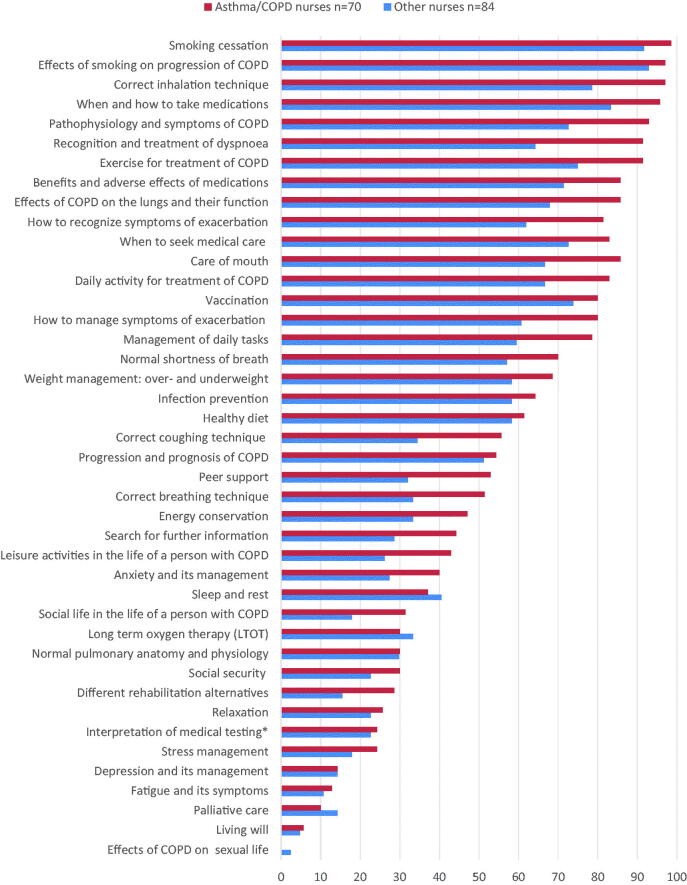
The regularity of self-management education provided by asthma/COPD nurses (*n* = 70) and nurses who do not work as an asthma/COPD nurse (*n* = 84). *Interpretation of the results of spirometry and other physical measurements.

**Figure 4.**
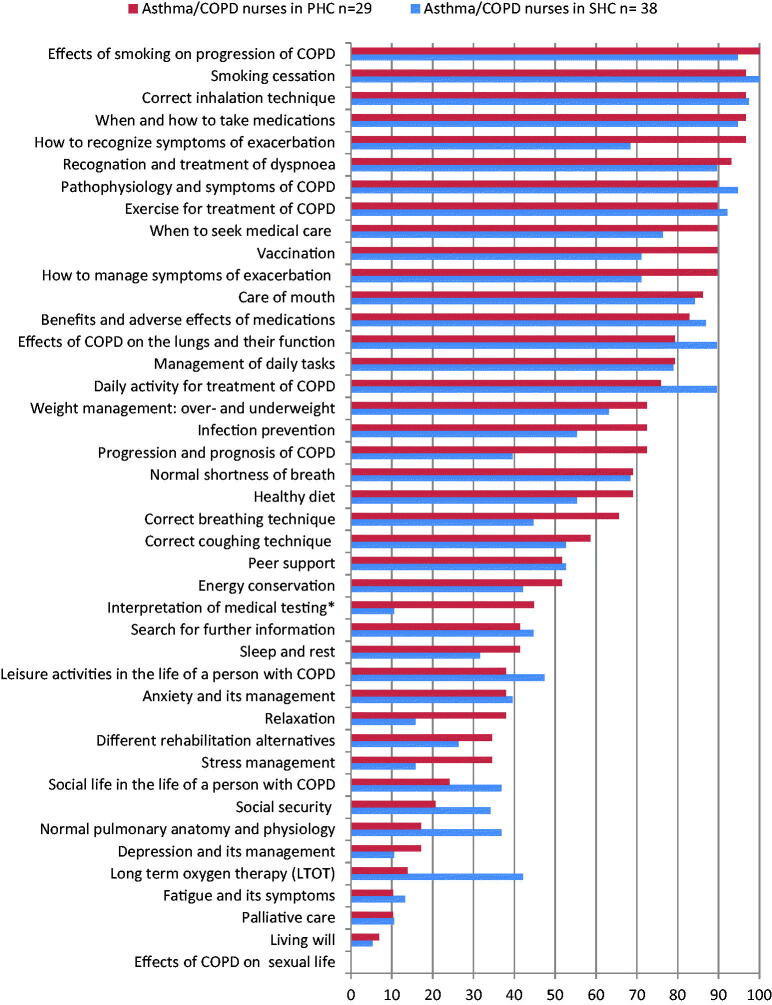
The regularity of self-management education provided by asthma/COPD nurses in PHC (*n* = 29) and in SHC (*n* = 38). *Interpretation of the results of spirometry and other physical measurements.

The patient education provided by the asthma/COPD nurses (*n* = 70) was offered more regularly than education provided by the other nurses (*n* = 84) (Figure 3). When comparing the patient education provided by the asthma/COPD nurses in PHC and SHC, there were differences in the regularity of education, depending on the subject (Figure 4).

## Discussion

### Principal findings

According to this study, self-management education regularly included medical issues regarding COPD treatment, such as smoking cessation, exercise and pharmacological treatment. However, psychosocial issues vital for coping with the disease, such as psychological well-being, stress and fatigue, were often ignored. Doctors and nurses tended to have their own scopes regarding the contents of patient education. Education in PHC seemed to be less systematic than education in SHC. The asthma/COPD nurses provided education more regularly than the other nurses.

### Strengths and weaknesses of the study

The respondents were obtained using purposeful sampling, because it was impossible to include the doctors and nurses from all the health centres in Finland (*n* = 160) in this study. The PHC sample was representative based on the healthcare organisations’ size and the location in the country but included only nine PHC centres (6% of all PHC centres). The sample of SHC units covered all Finnish pulmonary outpatient clinics in continental Finland, resulting in good response rates from doctors (73% of units) and nurses (90% of units). Consequently, the PHC sample size of this study does not support generalisability. However, the results are indicative, and they add understanding and give reasons for further development and research.

Due to a variation among COPD patients in real life, it may have been difficult to evaluate patient education on a general level. Furthermore, the original survey included additional questions regarding the local arrangements of COPD patients’ care, which will be reported later. Thus, the questionnaire may have been too long and time-consuming for some potential respondents.

### Findings in relation to other studies

The results of our study showed that doctors and nurses emphasise partially different contents of COPD self-management education, which is a natural consequence of doctors’ and nurses’ education and job descriptions. Thus, good self-management education requires that both parties know their own responsibilities in patient education and that roles of doctors and nurses is accepted and consistent throughout the whole organisation. Otherwise, it is possible that patient education is coincidental and COPD patients do not receive the education they need. This is noteworthy especially in Finnish PHC, where nurses’ responsibility for COPD patients’ self-management education seems to be extensive.

In the present study, only a few doctors and nurses regularly educated COPD patients on depression, anxiety, stress or social life. This might be due to lack of knowledge or consultation time, making a holistic approach unrealistic [14–16]. However, psychosocial well-being is interrelated with quality of life [17] and the patients’ motivation to engage in self-management [18], and therefore needs the attention of healthcare professionals.

Despite good management, COPD is highly symptomatic, especially in the advanced phases of the disease [19], and fatigue is one of the most prevalent symptoms [20,21]. Our results are consistent with previous studies showing that fatigue often goes unnoticed by family members as well as healthcare professionals [22,23].

In this study, palliative care was rarely included in the self-management education. Discussing palliative care can be ignored, for example, due to uncertainty regarding the COPD prognosis [24] or a COPD patient’s unwillingness to discuss palliative care [25]. However, discussion about death and palliative care might alleviate COPD patients’ fears and offer them an opportunity to plan the rest of their lives [26]. It is noteworthy that COPD patients are less likely to receive palliative care compared with cancer patients [4]. Thus, the duty of healthcare professionals should be first to provide an opportunity to discuss palliative care early enough and, thereafter, an opportunity for such care.

It is obvious that the types of patients (e.g. GOLD stage, multimorbidity) whom healthcare professionals take care of affect the content of COPD education that is provided in PHC and SHC. In this light, some of the results can be seen as a natural consequence of individualised care. For instance, not all COPD patients need education in palliative care or living wills, and the recommended COPD care is often compromised and modified because of multiple health or social issues.

In Finland, PHC is in charge of the early detection, diagnosis and management of COPD in all GOLD stages, except for exceptionally young patients or those who have very severe COPD, problems with diagnosis, or those who need LTOT [8]. COPD patients are often multi-morbid, and COPD, as one of the patient’s diseases and complaints, can be deprioritised under the general practitioners’ time constraints [15]. It is noteworthy that in Finnish PHC, only one in two patients meets the same doctor when visiting a health centre [27]. Also, a general practitioner’s knowledge of COPD and adherence to current guidelines may be insufficient [14]. In light of these facts, the irregularities in patient education seen in this study in PHC are a cause for concern.

According to our study, PHC nurses seemed to be responsible for a larger scope of COPD self-management education than SHC nurses, presumably with lesser support from PHC doctors compared with SHC consultants. Previous research indicates that PHC nurses may lack the knowledge and skills to carry out satisfactory care for COPD patients [16]. Lack of support from co-workers can result in the insecurity of nurses and hence make it difficult for them to develop COPD patients´ education [28]. However, previous findings show that with sufficient training, nurse-led asthma/COPD clinics in PHC may result in fewer exacerbations and hospitalisations for COPD patients [29].

In our study, COPD education provided by Finnish asthma/COPD nurses seemed to contain many issues regarded as essential for COPD patients’ self-management. Furthermore, the education provided by PHC’s asthma/COPD nurses seemed to be at least as regular as SHC’s asthma/COPD nurses. Thus, our results are consistent with others in showing that establishing nurse-led asthma/COPD clinics in PHC could be beneficial while also improving patient education practices that enhance patients’ ability to cope with the disease [29].

## Conclusions and implications for practice

Each COPD patient should have a regular opportunity to discuss issues vital for self-management with both a doctor and a nurse. Thus, the role of doctors and nurses, especially in PHC, should be considered to ensure that there is no gap in COPD patients’ education. Whether in SHC or PHC, patient education should be provided with a holistic approach by doctors and nurses who have up-to-date knowledge and the skills suitable for COPD care. Healthcare professionals’ continuing medical education should cover not only the medical but also the psychosocial aspects of coping with the disease. The patient education provided by asthma/COPD nurses, especially in PHC, seems to be encouraging, which should be acknowledged while improving patient education in PHC. Further research should focus on possible gaps between COPD patients’ needs and the COPD education provided by healthcare professionals.

## Supplementary Material

Supplemental Material

## References

[1] Lozano R, Naghavi M, Foreman K, et al. Global and regional mortality from 235 causes of death for 20 age groups in 1990 and 2010: a systematic analysis for the Global Burden of Disease Study 2010. Lancet. 2012;15:2095–2128. 10.1016/S0140-6736(12)61728-0 PMC10790329 23245604

[2] Forey BA, Thornton AJ, Lee PN. Systematic review with meta-analysis of the epidemiological evidence relating smoking to COPD, chronic bronchitis and emphysema. BMC Pulm Med. 2011;11(1):36. 21672193 10.1186/1471-2466-11-36 PMC3128042

[3] Pooler A, Beech R. Examining the relationship between anxiety and depression and exacerbations of COPD which result in hospital admission: a systematic review. Int J Chron Obstruct Pulmon Dis. 2014;29:315–330. 10.2147/COPD.S53255 PMC3974694 24729698

[4] GOLD. 2018. Global initiative for chronic obstructive lung disease. Global strategy for the diagnosis, management, and prevention of chronic obstructive pulmonary disease. 2018 Report. [cited 2018 Nov]. Available from: http://goldcopd.org/wp-content/uploads/2017/11/GOLD-2018-v6.0-FINAL-revised-20-Nov_WMS.pdf

[5] Zwerink M, Brusse-Keizer M, van der Valk PD, et al. Self-management for patients with chronic obstructive pulmonary disease. Cochrane Database Syst Rev. 2014;CD002990. 24665053 10.1002/14651858.CD002990.pub3 PMC7004246

[6] Lorig KR, Holman H. Self-management education: history, definition, outcomes, and mechanisms. Ann Behav Med. 2003;26(1):1–7. 12867348 10.1207/S15324796ABM2601_01

[7] Bourbeau J, Nault D, Dang-Tan T. Self-management and behaviour modification in COPD. Patient Educ Couns. 2004;52(3):271–277. 14998597 10.1016/S0738-3991(03)00102-2

[8] Kankaanranta H, Harju T, Kilpelainen M, et al. Diagnosis and pharmacotherapy of stable chronic obstructive pulmonary disease: the Finnish guidelines. Basic Clin Pharmacol Toxicol. 2015;116(4):291–307. 25515181 10.1111/bcpt.12366 PMC4409821

[9] Hyland ME, Jones RC, Hanney KE. The lung information needs questionnaire: Development, preliminary validation and findings. Respir Med. 2006;100(10):1807–1816. 16524709 10.1016/j.rmed.2006.01.018

[10] White R, Walker P, Roberts S, et al. Bristol COPD Knowledge Questionnaire (BCKQ): testing what we teach patients about COPD. Chron Respir Dis. 2006;3(3):123–131. 16916006 10.1191/1479972306cd117oa

[11] Stoilkova A, Janssen DJ, Wouters EF. Educational programmes in COPD management interventions: a systematic review. Respir Med. 2013;107(11):1637–1650. 24012387 10.1016/j.rmed.2013.08.006

[12] Tan JY, Chen JX, Liu XL, et al. A meta-analysis on the impact of disease-specific education programs on health outcomes for patients with chronic obstructive pulmonary disease. Geriatr Nurs. 2012;33(4):280–296. 22595334 10.1016/j.gerinurse.2012.03.001

[13] National Institute for Health and Care Excellence (NICE). Chronic obstructive pulmonary disease overview. [cited 2016 August]. Available from: https://pathways.nice.org.uk/pathways/chronic-obstructive-pulmonary-disease 31211541

[14] Sandelowsky H, Natalishvili N, Krakau I, et al. COPD management by Swedish general practitioners – baseline results of the PRIMAIR study. Scand J Prim Health Care. 2018;36(1):5–13. 29334861 10.1080/02813432.2018.1426148 PMC5901441

[15] Sandelowsky H, Hylander I, Krakau I, et al. Time pressured deprioritization of COPD in primary care: a qualitative study. Scand J Prim Health Care. 2016;34(1):55–65. 26849465 10.3109/02813432.2015.1132892 PMC4911027

[16] Berland A, Bentsen SB. Patients with chronic obstructive pulmonary disease in safe hands: an education programme for nurses in primary care in Norway. Nurse Educ Pract. 2015;15(4):271–276. 25881490 10.1016/j.nepr.2015.03.003

[17] Blakemore A, Dickens C, Guthrie E, et al. Depression and anxiety predict health-related quality of life in chronic obstructive pulmonary disease: systematic review and meta-analysis. Int J Chron Obstruct Pulmon Dis. 2014;9:501–512. 24876770 10.2147/COPD.S58136 PMC4035108

[18] Russell S, Ogunbayo OJ, Newham JJ, et al. Qualitative systematic review of barriers and facilitators to self-management of chronic obstructive pulmonary disease: views of patients and healthcare professionals. NPJ Prim Care Respir Med. 2018;28(1):2. 29343739 10.1038/s41533-017-0069-z PMC5772437

[19] Joshi M, Joshi A, Bartter T. Symptom burden in chronic obstructive pulmonary disease and cancer. Curr Opin Pulm Med. 2012;18(2):97–103. 22262138 10.1097/MCP.0b013e32834fa84c

[20] Christensen VL, Holm AM, Cooper B, et al. Differences in symptom burden among patients with moderate, severe, or very severe chronic obstructive pulmonary disease. J Pain Symptom Manage. 2016;51(5):849–859. 26899820 10.1016/j.jpainsymman.2015.12.324

[21] Eckerblad J, Todt K, Jakobsson P, et al. Symptom burden in stable COPD patients with moderate or severe airflow limitation. Heart Lung. 2014;43(4):351–357. 24856227 10.1016/j.hrtlng.2014.04.004

[22] Celli B, Blasi F, Gaga M, et al. Perception of symptoms and quality of life – comparison of patients’ and physicians’ views in the COPD MIRROR study. COPD. 2017;27:2189–2196. 10.2147/COPD.S136711 PMC5538543 28794623

[23] Stridsman C, Lindberg A, Skar L. Fatigue in chronic obstructive pulmonary disease: a qualitative study of people’s experiences. Scand J Caring Sci. 2014;28(1):130–138. 23517049 10.1111/scs.12033

[24] Curtis JR. Palliative and end-of-life care for patients with severe COPD. Eur Respir J. 2008;32(3):796–803. 17989116 10.1183/09031936.00126107

[25] Jones I, Kirby A, Ormiston P, et al. The needs of patients dying of chronic obstructive pulmonary disease in the community. Fam Pract. 2004;21(3):310–313. 15128695 10.1093/fampra/cmh317

[26] Gardiner C, Gott M, Small N, et al. Living with advanced chronic obstructive pulmonary disease: patients concerns regarding death and dying. Palliat Med. 2009;23(8):691–697. 19825897 10.1177/0269216309107003

[27] Raivio R, Jaaskelainen J, Holmberg-Marttila D, et al. Decreasing trends in patient satisfaction, accessibility and continuity of care in Finnish primary health care – a 14-year follow-up questionnaire study. BMC Fam Pract. 2014;15(1):98. 24885700 10.1186/1471-2296-15-98 PMC4030039

[28] Zakrisson A, Hägglund D. The asthma/COPD nurses’ experience of educating patients with chronic obstructive pulmonary disease in primary health care. Scand J Caring Sci. 2010;24(1):147–155. 19691488 10.1111/j.1471-6712.2009.00698.x

[29] Lisspers K, Johansson G, Jansson C, et al. Improvement in COPD management by access to asthma/COPD clinics in primary care: data from the observational PATHOS study. Respir Med. 2014;108(9):1345–1354. 25002194 10.1016/j.rmed.2014.06.002

