# Supplementary material for: Psychosocial issues need more attention in COPD self-management education
Source: Scand J Prim Health Care. 2020 Feb 6;38(1):47–55. doi: 10.1080/02813432.2020.1717087 (PMC7054946; doi:10.1080/02813432.2020.1717087)
Supplement: Supplemental Material [file IPRI_A_1717087_SM6506.pdf]

The questionnaire for doctors and nurses

The questions for doctors and nurses:

1. How old are you? _____yrs

2. How long have you worked as a nurse? ____yrs

   How long have you worked as a doctor? ____yrs

3. Do you work at an outpatient clinic

   O in specialised healthcare O in primary healthcare

4. How long have you treated COPD patients? _____yrs

5. Does an asthma/COPD nurse work in the same outpatient clinic where you work?

   O Yes O No

The question for nurses:

6. Do you work as an asthma/COPD nurse yourself?

   O Yes O No

For the following contents of patient education, consider your practice on a general level. How often do you include each of the contents in your education with COPD patients? The response options are: Education is provided 1) regularly (= usually, when you meet a COPD patient); 2) sometimes; 3) on the request of the patient; or 4) education is not provided at all. You can choose only one option.

	regularly	sometimes	on the request of the patient	education is not provided at all
Normal pulmonary anatomy and physiology	O	O	O	O
Pathophysiology and symptoms of COPD	O	O	O	O
Effects of COPD on the lungs and their function	O	O	O	O
Progression and prognosis of COPD	O	O	O	O
Interpretation of medical testing	O	O	O	O
Effects of smoking on progression of COPD	O	O	O	O
Smoking cessation	O	O	O	O
When and how to take medications	O	O	O	O

Benefits and adverse effects of medications	O	O	O	O
Correct inhalation technique	O	O	O	O
Care of mouth	O	O	O	O
Long term oxygen therapy (LTOT)	O	O	O	O
Importance of exercise for treatment of COPD	O	O	O	O
Importance of daily activity for treatment of COPD	O	O	O	O
Management of daily tasks	O	O	O	O
Energy conservation	O	O	O	O
Different rehabilitation alternatives	O	O	O	O
Vaccination	O	O	O	O
Infection prevention	O	O	O	O
Healthy diet	O	O	O	O
Weight management: over- and underweight	O	O	O	O
Normal shortness of breath	O	O	O	O
Recognition and treatment of dyspnoea	O	O	O	O
How to recognize symptoms of exacerbation	O	O	O	O
How to manage symptoms of exacerbation	O	O	O	O
When to seek medical care	O	O	O	O
Correct coughing technique (how to cough up phlegm)	O	O	O	O
Correct breathing technique	O	O	O	O
Anxiety and its management	O	O	O	O
Depression and its management	O	O	O	O
Stress management	O	O	O	O
Fatigue and its symptoms	O	O	O	O
Importance of leisure activities in the life of a person with COPD	O	O	O	O

Importance of social life in the life of a person with COPD	O	O	O	O
Effects of COPD on sexual life	O	O	O	O
Sleep and rest	O	O	O	O
Relaxation	O	O	O	O
Peer support	O	O	O	O
Search for further information	O	O	O	O
Social security (e.g. home help service, mobility, livelihood, taxation)	O	O	O	O
Palliative care	O	O	O	O
Living will	O	O	O	O
